# Hypoxia-induced myeloid derived growth factor promotes hepatocellular carcinoma progression through remodeling tumor microenvironment

**DOI:** 10.7150/thno.49327

**Published:** 2021-01-01

**Authors:** Xu Wang, Jie Mao, Tao Zhou, Xingyi Chen, Haoyang Tu, Jinyan Ma, Yixuan Li, Yushi Ding, Yong Yang, Hongxi Wu, Xinying Tang

**Affiliations:** 1 State Key Laboratory of Natural Medicines, China Pharmaceutical University, Nanjing, Jiangsu 211198, PR China.; 2 Center for New Drug Safety Evaluation and Research, China Pharmaceutical University, Nanjing, Jiangsu 211198, PR China.

**Keywords:** MYDGF, Hepatocellular carcinoma, Cancer stem cell, Tumor inflammatory microenvironment, Tumor-associated macrophages

## Abstract

**Purpose:** Exploring and studying the novel target of hepatocellular carcinoma (HCC) has been extremely important for its treatment. The principal objective of this project is to investigate whether myeloid derived growth factor (MYDGF) could accelerate the progression of HCC, and how it works.

**Methods:** Cell proliferation, clonal formation, sphere formation and xenograft tumor experiments were used to prove the critical role of MYDGF in HCC progression. Tumor angiogenesis, immune cell infiltration, macrophage chemotaxis and inflammatory cytokines detection were utilized to clarify how MYDGF remodeled the tumor microenvironment (TME) to accelerate the progress of HCC.

**Results:** Here, we reported a secretory protein MYDGF, which could be induced by hypoxia, was significantly upregulated in HCC and associated with poor clinical outcomes. Using bioinformatics and experimental approaches, we found that MYDGF promotes cell proliferation *in vitro* and *in vivo* through a mechanism that might involve enhanced self-renewal of liver CSCs. Furthermore, MYDGF can also promote tumor angiogenesis, induce macrophages to chemotaxis into tumor tissue, and then release various inflammatory cytokines, including IL-6 and TNF-α, which ultimately aggravate inflammation of tumor microenvironment and accelerate HCC progression.

**Conclusions:** We provided evidence that MYDGF could directly affect the self-renewal of liver CSCs, and indirectly aggravate the inflammatory microenvironment to accelerate the progression of HCC.

## Introduction

Hepatocellular carcinoma (HCC) is highly prevalent worldwide and characterized by extremely poor prognosis 1, 2. In China, HCC only has a 5-year survival rate of 14.1% 3 and a recurrence rate of about 70%. Treatment for HCC are limited, especially for patients with advanced disease who are not eligible for curative hepatectomy or hepatic transplantation. Sorafenib, a molecular-targeted drug used to against advanced HCC, has a partial response of only 2.2% and median overall survival (OS) of 9.2 months 4-6. In recent years, immunotherapy has overturned the way of cancer therapy 7. However, due to the immunosuppressive microenvironment and disordered inflammatory state in HCC, the efficiency of cancer immune response is not satisfied 8, 9. An ideal immunotherapeutic approach for HCC could promote a sustained increase of functional immune effector cells in the tumor through remodeling tumor microenvironment 10, 11. Understanding the underline mechanism of immunosuppression and inflammatory microenvironment disorder in HCC is essential for the development of rational treatment strategies.

There is growing evidence that supports the role of the tumor microenvironment in the development and progression of hepatocellular carcinoma 12. The tumor microenvironment (TME) is composed of tumor cells, stromal cells, vascular system, extracellular matrix, infiltrating immune cells and secreted proteins 13, 14. A complex array of interactions among cells within the TME is regulated by cancer cells for their own benefits and is facilitated by various secreted proteins, such as growth factors, cytokines, and tumor-derived proteins. Tumor cells could attract and activate macrophages by secreting various proteins (VEGF, PDGF, TGF-β, CCL2, and M-CSF) 15-17. Moreover, cytokines such as IL-6 and TNF-α also plays an important role in HCC progression. IL-6 is the major cytokine that stimulates proliferation of tumor cells, and it inhibits apoptosis via activation of the STAT3 signaling pathway 18 TNF-α could activate NF-κB and AKT signaling cascades and stimulate tumor progression 19. So, it is extremely important to investigate secreted proteins in the progression of HCC and their effects on the TME.

Myeloid-derived growth factor (MYDGF) is a 17-kDa secretory protein encoded by open reading frame 10 on human chromosome 19 20, which was reported to be highly expressed in synovial fluid during arthritis, suggesting a correlation between its secretion and joint inflammation 21. In addition, MYDGF was overexpressed in AFP-positive HCC samples. Overexpression MYDGF in AFP-negative HCC cells could promote cell proliferation* in vitro*, while knockdown suppressed cell cycle progression 22. This gene has also been reported to promote repair following myocardial infarction as well as cardiac myocyte survival and normal angiogenesis 23. Currently, the potential role of MYDGF in HCC progression and tumor microenvironment remains largely unknown.

Here, we investigate the mechanism by which MYDGF promotes the progression of HCC and how it affects the tumor microenvironment.

## Results

### MYDGF is highly expressed in HCC and associated with poor prognosis

**Figure 1:**
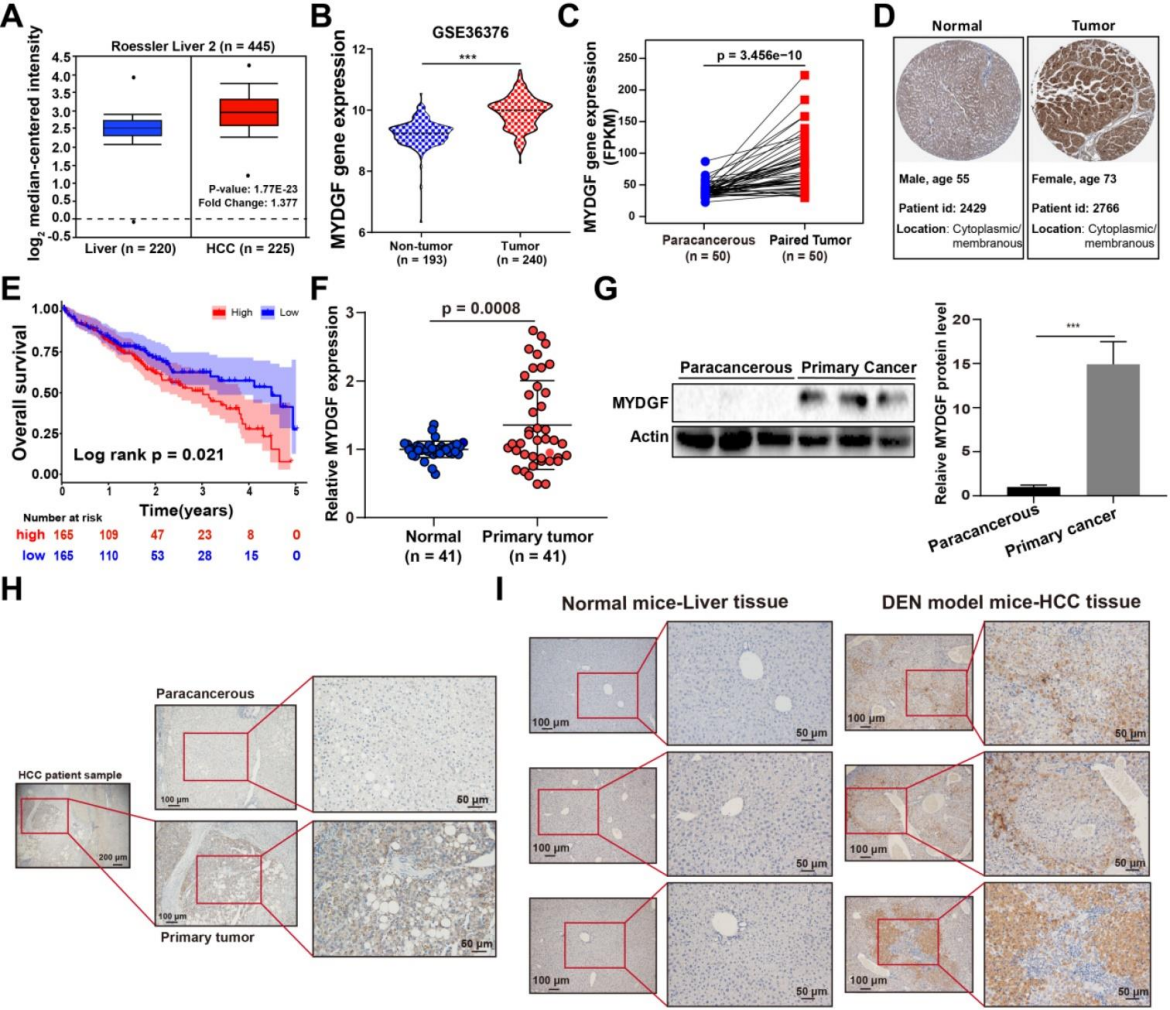
** MYDGF is highly expressed in HCC and correlates with poor prognosis.** (**A**) MYDGF gene expression in Roessler's liver datasets from Oncomine database. (**B**) MYDGF gene expression analysis in GSE36376 datasets from GEO database containing 193 non-tumor samples and 240 HCC tumor samples. (**C**) MYDGF gene expression in 50 paired normal and HCC samples from TCGA database. (**D**) Protein level of MYDGF in normal liver and HCC sample from ProtienAtlas database. (**E**) 5-year overall survival rate between MYDGF high and low expression groups. (**F**) mRNA expression of MYDGF in human normal tissue and primary HCC samples by qPCR (n = 41). (**G**) Protein level of MYDGF in normal and primary HCC samples, 3 samples in each group was showed. (**H**) Level of MYDGF protein in paracancerous and primary HCC tumor samples as detected by immunohistochemistry. (I) Protein level of MYDGF in normal mouse liver tissues and DEN-induced mouse HCC samples as detected by immunohistochemistry.

To assess the correlation between MYDGF and HCC development, we used the Oncomine and GEO database to analyze MYDGF mRNA expression. We found that MYDGF expression levels are significantly higher in HCC tissues than normal ones (Oncomine, Figure 1A, Figure S1A, *p* < 0.01, GEO, Figure 1B, Figure S1B-D, *p* < 0.001). This observation was further validated in paired HCC data from the cancer genome atlas (TCGA) (Figure 1C, *p* < 0.0001). Next, we confirmed the above results at the translational level on ProteinAtlas database (Figure 1D). Overall survival analysis of HCC patients from TCGA database showed that survival time in MYDGF^high^ group was noticeably shorter relative to the lower ones (High and low expression groups were based on median MYDGF expression in all HCC samples from TCGA database). The MYDGF^low^ group exhibited a median survival of 53.35 months vs 35.74 months for the MYDGF^high^ group (Figure 1E). Prognosis-related gene expression levels are often closely related to clinical stage. Here, we observed that MYDGF expression was higher in M1 stage relative to M0 stage, suggesting MYDGF accumulation with tumor malignant and metastatic state (Figure S1E).

Next, we validated the above bioinformatic analysis both* in vitro* and *in vivo*. Paired HCC primary tumor and paracancerous samples revealed that MYDGF was higher in HCC both transcriptionally and translationally (Figure 1F-G, *p* < 0.001). However, MYDGF expression was barely elevated in cirrhotic tissues, indicating that suggesting that it is highly specific for the malignant stage (Figure S1G). IHC analysis showed that overexpressed MYDGF mainly localizes in the cytoplasm and membrane, consistent with its secretory nature (Figure 1H). Results obtained from a classic diethylnitrosamine (DEN)-induced mouse HCC model was similar to the clinical samples (Figure 1I). These results show that MYDGF is generally up-regulated in HCC and is significantly related to poor prognosis.

### MYDGF could directly promote proliferation of HCC *in vitro* and *in vivo*

**Figure 2:**
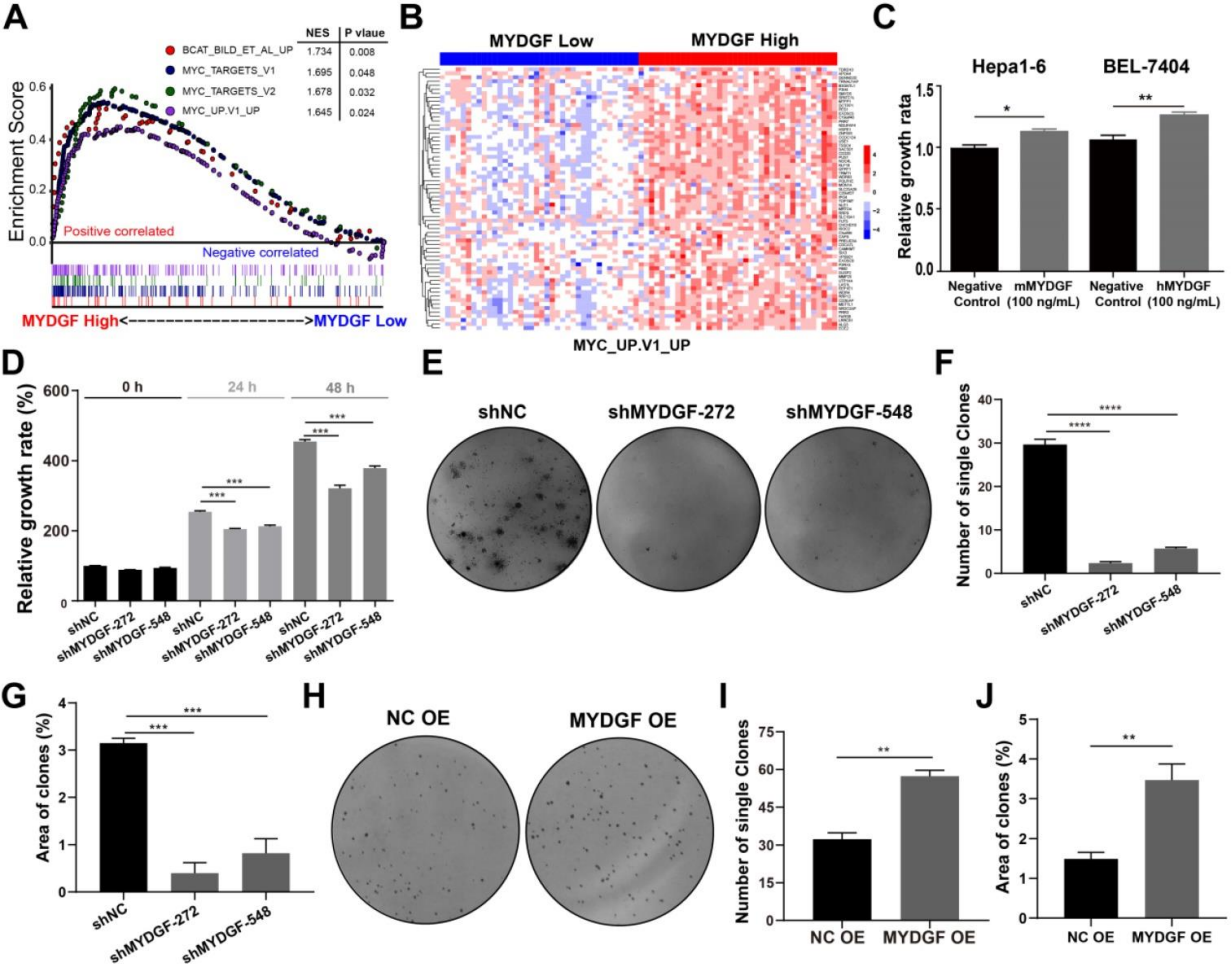
** MYDGF promotes cell proliferation *in vitro*.** (**A**) Single gene GSEA enrichment analysis indicating that four proliferation related signatures were significantly enriched in MYDGF high expression group. Normalized enrichment score (NES), nominal P-value were shown in the plot. (**B**) The expression pattern of MYDGF in the high and low groups of one enriched signature “MYC_UP. V1_UP”. (**C**) The relative proliferation rate of murine Hepa1-6 and human BEL-7404 HCC cell lines cultured with 100 ng/mL recombinant murine and human MYDGF protein, respectively for 24 h by SRB. (**D**) The effects of MYDGF knockdown on the proliferation rate in Hepa1-6 cells by SRB. (**E**) The effect of MYDGF knockdown on clone formation of Hepa1-6 cells. (**F**) Clone numbers and (**G**) area fraction of MYDGF knockdown counted. (**H**) The clone formation assay based on MYDGF overexpressed cells. (**I-J**) Clone numbers and area fraction of MYDGF overexpression were counted.

Next, we adopted a single-gene GSEA enrichment assay between MYDGF^high^ and MYDGF^low^ group to forecast the relationship between MYDGF and HCC. This analysis uncovered multiple proliferation-related pathways that positively correlated with the MYDGF^high^ group, including “HALLMARK MYC TARGETS V1” (*p* = 0.048), “HALLMARK MYC TARGETS V2” (*p* = 0.032), “MYC UP. V1 UP” (*p* = 0.024) and "BCAT BILD ET AL UP" (*p* = 0.008) (Figure 2A). Gene expression heatmap showed significantly different expression pattern between MYDGF^low^ and MYDGF^high^ group in four enriched signatures (Figure 2B and Figure S2A). These data suggested that MYDGF is closely related to cell proliferation.

**Figure 3:**
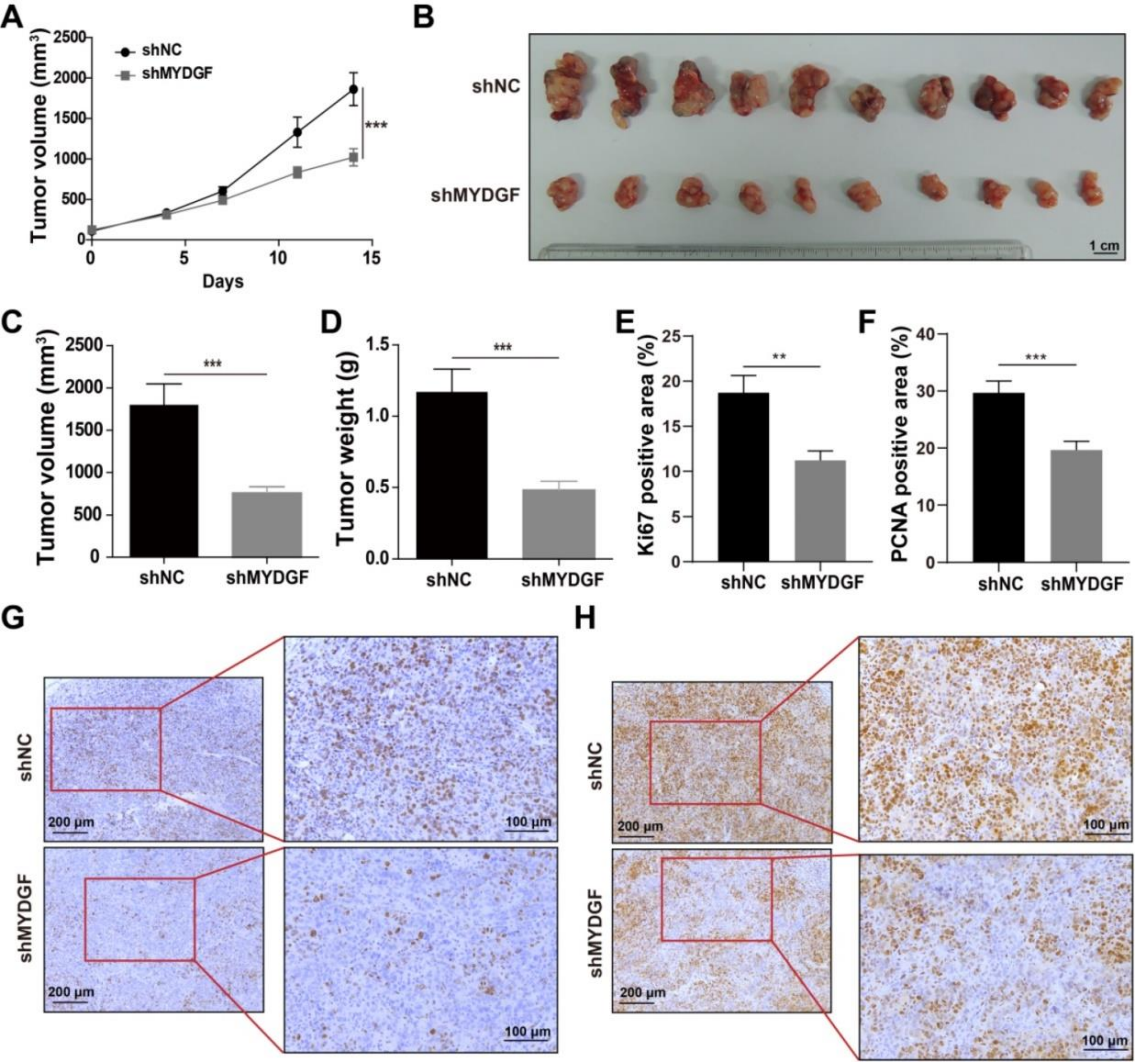
** MYDGF promotes proliferation *in vivo*.** (**A**) Tumor growth curve of MYDGF knockdown in C57/BL6 xenograft mice models. Tumor volume was calculated by the formula: 1/2 length*width^2^. (**B**) Tumor picture at the end of the experiment. Tumor volume (**C**) and weight (**D**) measured at the end of the experiment. Quantitative results of Ki67 (**E**) and PCNA (**F**) positive staining area percentage by immunohistochemistry. Immunohistochemistry staining of Ki67 (**G**) and PCNA (**H**).

To confirm the above assumption, we constructed the MYDGF overexpressing or knocking down in AFP-positive HCC cell lines (Figure S2B) and performed proliferation-related assays. Knockdown and overexpression efficiency were validated by qPCR or Western Blot (Figure S2C-D). Exogenous addition of recombinant MYDGF protein promoted Hepa1-6 and BEL-7404 proliferation (Figure 2C, *p* < 0.05) while knockdown suppressed cell growth around 30% at 48h (Figure 2D, *p* < 0.001). Furthermore, MYDGF knockdown in Hepa1-6 suppressed colony formation about 70% (*p* < 0.05) (Figure 2E-G). Overexpression promoted colony formation of BEL-7404 (Figure 2H-J, *p* < 0.001). We then xenotransplanted Hepa1-6-shMYDGF and Hepa1-6-shNC cells in C57BL/6 mice and monitored tumor growth. Tumor growth was significantly delayed to around 60% in the knockdown group by day 14 (Figure 3A-D, *p* < 0.001). At the end of the experiment, gene expression level of MYDGF in tumor tissues was confirmed, and the knockdown efficiency remained above 70% (Figure S2E). IHC analysis of Ki67 and PCNA in tumor tissues revealed that proliferation was markedly reduced in the MYDGF knockdown group (Figure 3G-H, *p* < 0.05). The percentage of ki67 and PCNA positive staining area was quantitatively counted to evaluate the proliferation inhibition effect (Figure 3E-F, *p* < 0.01). Taken together, these findings showed that MYDGF could promote HCC proliferation *in vitro* and* in vivo*.

### MYDGF could enhance self-renewal of liver CSCs

**Figure 4:**
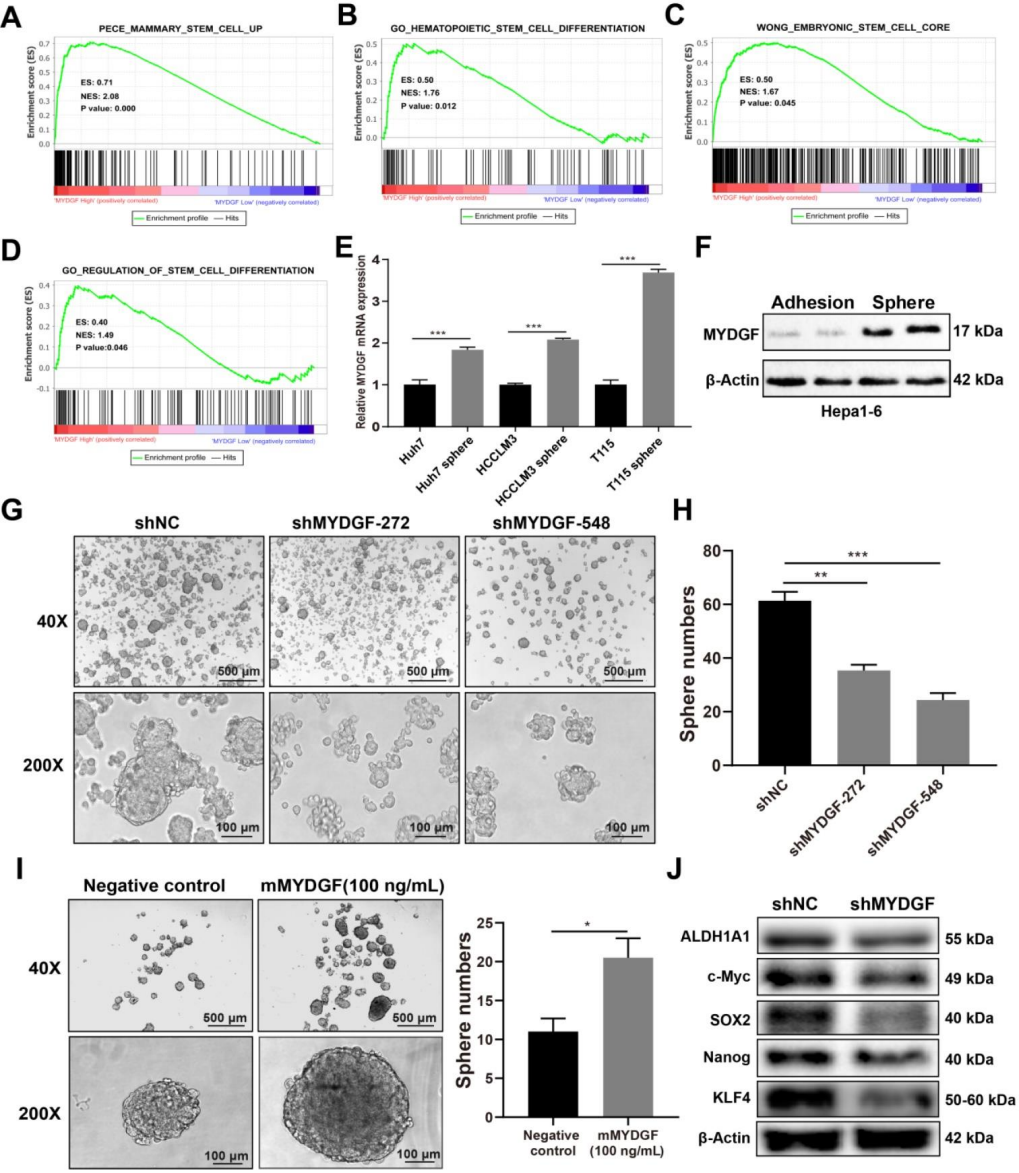
** MYDGF enhances self-renewal of liver CSCs.** (**A-D**) GSEA of stem cell related signatures in MYDGF high versus low samples from TCGA database. Normalized enrichment score (NES), nominal P-value is shown in each plot. (**E**) MYDGF mRNA expression in non-sphere and sphere human HCC cell lines. (**F**) MYDGF protein expression in non-sphere and sphere Hepa1-6 cell. (**G**) The effect of MYDGF knockdown on the sphere formation ability of Hepa1-6 cells. (**H**) Quantitatively count sphere numbers of Hepa1-6 after knocking down MYDGF. (**I**) Sphere formation and statistics of Hepa1-6 cells treated with murine recombinant MYDGF. (**J**) Expression of stemness-related proteins after MYDGF knocking down.

In previous experiments, we observed more significant inhibition* in vivo* as compared with *in vitro* assays. Therefore, we speculated that other factors might affect *in vivo* tumor growth. Previous studies suggested that MYDGF played a role in regeneration 24 while stem-like properties could accelerate tumor cell proliferation 25-29. We speculated that MYDGF might influence proliferation by promoting cancer stem-like properties in HCC. To address this possibility, we performed single gene GSEA analysis using stem related signatures as the background signature. Four signatures enriched in MYDGF^high^ group (Figure 4A-D, p < 0.05). Analyzing correlation between MYDGF and top 3 enriched genes from each signature, revealed a high positive correlation (Figure S3A). Among these genes, SEC61G exhibited the strongest correlation (Figure S3B, R = 0.66, p < 0.001), which was closely related to clonal expansion of stem cells 30. These data imply that MYDGF is closely linked to cell stemness.

We then investigated the effect of MYDGF in liver CSCs formation *in vitro*. qPCR revealed MYDGF was significantly upregulated in 3 human HCC cell line spheres relative to adherent ones (Figure 4E, *p* < 0.001). Similar observation was found by Western Blot analysis in Hepa1-6 spheres (Figure 4F). To assess whether MYDGF is a driver or passenger gene in CSCs formation, we induced sphere formation in Hepa1-6-shMYDGF cells and found the number and size of spheres significantly decreased (Figure 4G-H, *p* < 0.01), which was rescued by exogenous MYDGF (Figure 4I, *p* < 0.05). Western Blot showed that stemness-related proteins were significantly downregulated in MYDGF knockdown cells, including ALDH1A1, c-Myc, SOX2, Nanog and KLF4, which could partially explain the effect of MYDGF knockdown on spheres formation (Figure 4J). Additionally, we observed a significant positive correlation between the expression level of MYDGF and OCT4 in 3 HCC cell lines (Figure S3C-E, R > 0.8, *p* < 0.05). All these data indicating MYDGF could enhance self-renewal of liver CSCs.

### MYDGF could indirectly promote HCC development by remodeling TME

Previously, MYDGF has been reported to promote myocardial infarction repair via angiogenesis 23. Considering aberrant angiogenesis in tumor progression, we explored the role of MYDGF during this pathogenic process. We performed IHC for CD31 in xenograft tumors, which revealed significantly lower CD31 levels in MYDGF knockdown group (Figure S4A-B). *In vitro* tubulin formation experiment showed tubules formed by HUVEC cells were significantly enhanced by exogenous MYDGF (50 ng/mL) (Figure S4C-D, total branching length, *p* < 0.01, total meshes area, *p* < 0.01). These results indicated MYDGF can directly promote tumor angiogenesis of HCC.

**Figure 5:**
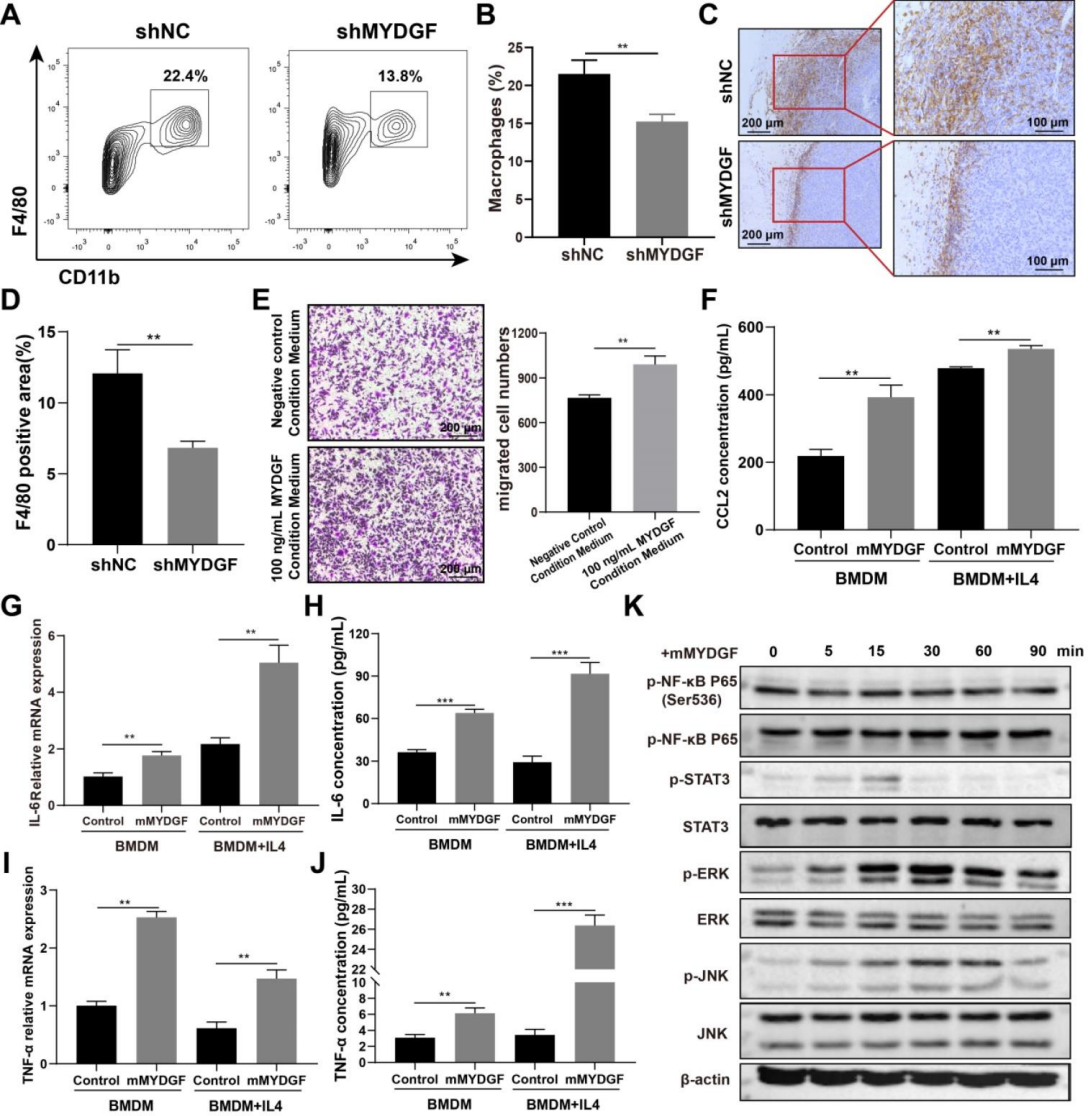
** MYDGF indirectly promoted HCC development by remodeling TME.** (**A**) The proportion of macrophage infiltration in tumor tissue detected by flow cytometry. (**B**) Quantitative of macrophage infiltration percentage. (**C**) Immunohistochemistry of F4/80 positive cells in tumor tissues. (**D**) Quantitative analysis of F4/80 positive cells in tumor tissues. (**E**) Transwell experiments to assess chemotaxis of MYDGF on macrophages. (**F**) CCL2 protein level in BMDM and M2-like macrophages cultured with 100 ng/mL MYDGF recombinant protein. IL-6 mRNA (**G**) and protein (**H**) level in BMDM and M2 type macrophages cultured with 100 ng/mL MYDGF recombinant protein. TNF-α mRNA (**I**) and protein (**J**) level in BMDM and M2 type macrophages cultured with 100 ng/mL MYDGF recombinant protein. (**K**) Expression of inflammation-related proteins in macrophages after culturing with 100 ng/mL murine MYDGF recombinant protein.

Tumorigenesis of HCC is usually associated with chronic inflammatory process. Therefore, we investigated the potential effect of MYDGF on HCC from this perspective. We repeated the Hepa1-6 xenograft models and analyzed the infiltrated immune profiles by flow cytometry. Here we found that the proportion of infiltrated TAM was reduced in MYDGF knockdown group (Figure 5A-B, *p* < 0.01). The results were further confirmed by F4/80 IHC staining (Figure 5C-D, *p* < 0.01). In addition, we also observed an upregulation tendency of CD3^+^CD8^+^ T cells in MYDGF knockdown group (Figure S4E-F, *p* = 0.072). To uncover the underlying mechanism, we conducted macrophage chemotaxis experiments *in vitro*. Exogenous administration of 100 ng/mL MYDGF could not directly induce macrophage chemotaxis, while the supernatant from MYDGF treated macrophages could enhance BMDM infiltration (Figure 5E, *p* < 0.01). Moreover, we analyzed the chemokine expression and found that the CCL2 secretion could be elevated by MYDGF treatment, which might explain the above phenomenon (Figure 5F, S5G, *p* < 0.05).

The next question is how MYDGF-induced TAM remodel TME. Therefore, we used 100 ng/mL MYDGF to stimulate BMDM and M2 type macrophages *in vitro* and determine the cytokine expression. The mRNA and protein expression of IL-6 and TNF-α were increased in BMDM and M2 like macrophage when treated with MYDGF (Figure 5G-J, *p* < 0.01). The characteristic genes of M2 type macrophages including Arg1, Retnla and IL-10 both showed an upward trend in M2 type macrophages but not BMDM (Figure S4H-J, *p* < 0.01). To assess the underlying mechanism, we examined key components in macrophage inflammatory signaling pathways and observed that MAPK (Erk1/Erk2), JNK and STAT3 were phosphorylated in 15 min after MYDGF addition (Figure 5K), implying MAPK and STAT3 signaling activation. These results demonstrated that MYDGF could remodel tumor microenvironment by enhancing tumor angiogenesis, macrophage chemotaxis and inflammatory cytokines release.

### Hypoxia could induce MYDGF upregulation in HCC

**Figure 6:**
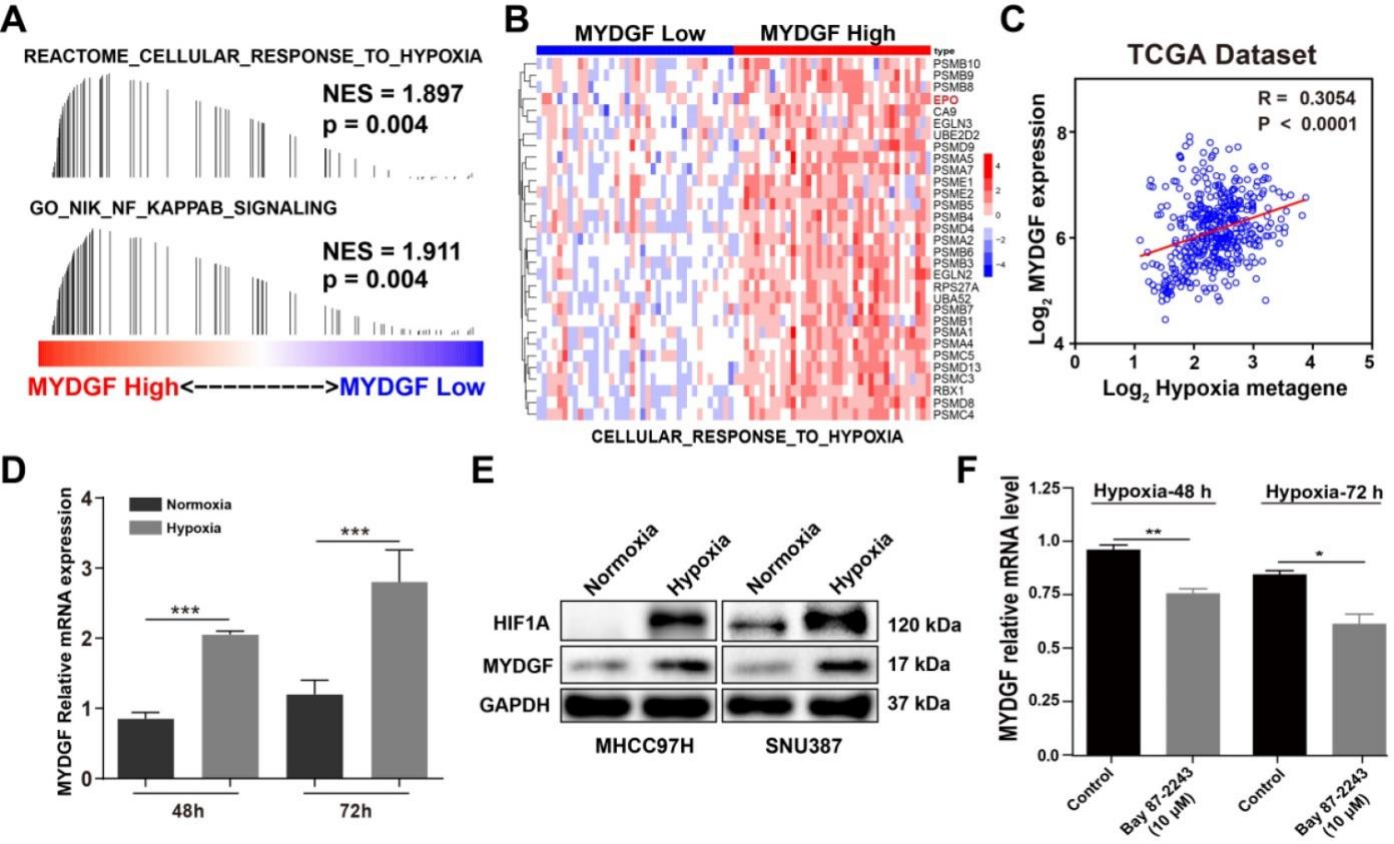
** Hypoxia induces MYDGF expression in HCC.** (**A**) Single gene GESA revealed that hypoxia and its related signaling pathways were enriched in the high MYDGF expression group. The color bars indicate that genes are ranked according to the high and low expression of MYDGF (Red represents high MYDGF expression, Blue represents low MYDGF expression). The vertical line indicates genes in the feature set, and the position of the vertical line indicates genes that appears in the ranking gene list position, the height of the bars indicates the running GSEA enrichment score. (**B**) Gene expression heat map of “CELLULAR RESPONSE TO HYPOXIA” signature in MYDGF high and low groups. (**C**) Correlation between MYDGF and hypoxia metagenes in TCGA liver cancer dataset. (**D**) MYDGF mRNA expression during normoxia and hypoxia. (**E**) MYDGF protein level in two HCC cell lines under hypoxia condition. (**F**) MYDGF mRNA expression in cell treated with HIF inhibitor Bay 87-2243 and exposed to hypoxia for 48 and 72 hours.

Next, we examined how MYDGF was upregulated in HCC by GESA analysis. We found that "CELLULAR RESPONSE TO HYPOXIA" and “NF-κB SINALING” gene signatures were also significantly enriched in the MYDGF^high^ group (Figure 6A). Gene expression heatmap analysis revealed that the hypoxia and NF-κB signaling-related genesets were significantly elevated in MYDGF^high^ group (Figure 6B). Hypoxia-inducible factor-1α (HIF-1α) could regulate erythropoietin (EPO) gene activity according to oxygen concentration and adapt to physiological reaction such as hypoxia metabolism 31. In "CELLULAR RESPONSE TO HYPOXIA" signature, EPO was highly expressed in the MYDGF high expression group than the lower ones (Figure S5A, *p* < 0.01). We further analyzed the correlation between MYDGF and previously reported hypoxia metagenes with the TCGA LIHC dataset 32 and observed a > 0.3 correlation (*p* < 0.0001), suggesting that MYDGF was positively correlated with hypoxia metagenes (Figure 6C). Evaluation of MYDGF expression in cells cultured under hypoxic (1% O_2_) vs normoxic (21% O_2_) conditions revealed significantly higher MYDGF levels under hypoxia (Figure 6D), this result could be further demonstrated at the translation level (Figure 6E). Upregulated MYDGF expression could also be reversed by HIF inhibitor Bay 87-2243 (Figure 6F). Other possible accelerators that mimicked the HCC microenvironment were excluded (Figure S5B-C). These data suggest that hypoxia may be associated with the overexpression of MYDGF in HCC.

## Discussion

Based on microarray analysis from GEO and TCGA datasets, MYDGF was highly expressed in HCC and negatively correlated with prognosis. We also evaluated mRNA and protein level of MYDGF in human HCC and classically recognized mouse HCC samples. Collectively, our data indicated a critical role of MYDGF in HCC. *In vitro* and *in vivo* experiments have confirmed that MYDGF could promote the proliferation of AFP-positive HCC cells, probably through a mechanism that enhance cell self-renewal, which had not been reported before.

In addition, our research showed that MYDGF could promote tumor angiogenesis. Tumorigenesis depends not only on tumor cell proliferation, but also on the complex inflammatory microenvironments around cancer cells. Tumor-promoting inflammatory cells such as macrophages could play essential roles in tumor inflammatory microenvironment. Multiple studies have demonstrated that inflammation could promote tumorigenesis 33. Epidemiological studies indicated that inflammation-independent cancers also have inflammatory microenvironments. Their main features include leukocyte infiltration (mainly macrophages), presence of inflammatory cytokines (including IL-6 and TNF-α), and angiogenesis. Elevated levels of IL-6 are usually associated with poor survival outcome in many cancers 34, 35. Tumor-associated macrophages could produce IL-6, which would promote expansion of CSCs and tumorigenesis 36. Meanwhile, tumor necrosis factor alpha (TNF-α) is mainly secreted by active macrophages and monocytes, is a pleiotropic cytokine. TNF-α released by M2 macrophages can promote epithelial-mesenchymal transition and cancer stemness of HCC 37. Here, we found that MYDGF could induce macrophages chemotaxis and secretion of inflammatory cytokines by macrophages, indicating that MYDGF might remodel the tumor microenvironment to accelerate HCC progression. Furthermore, we elucidated hypoxia as the major cause of MYDGF upregulation, which can be reversed by HIF inhibitors.

**Figure 7:**
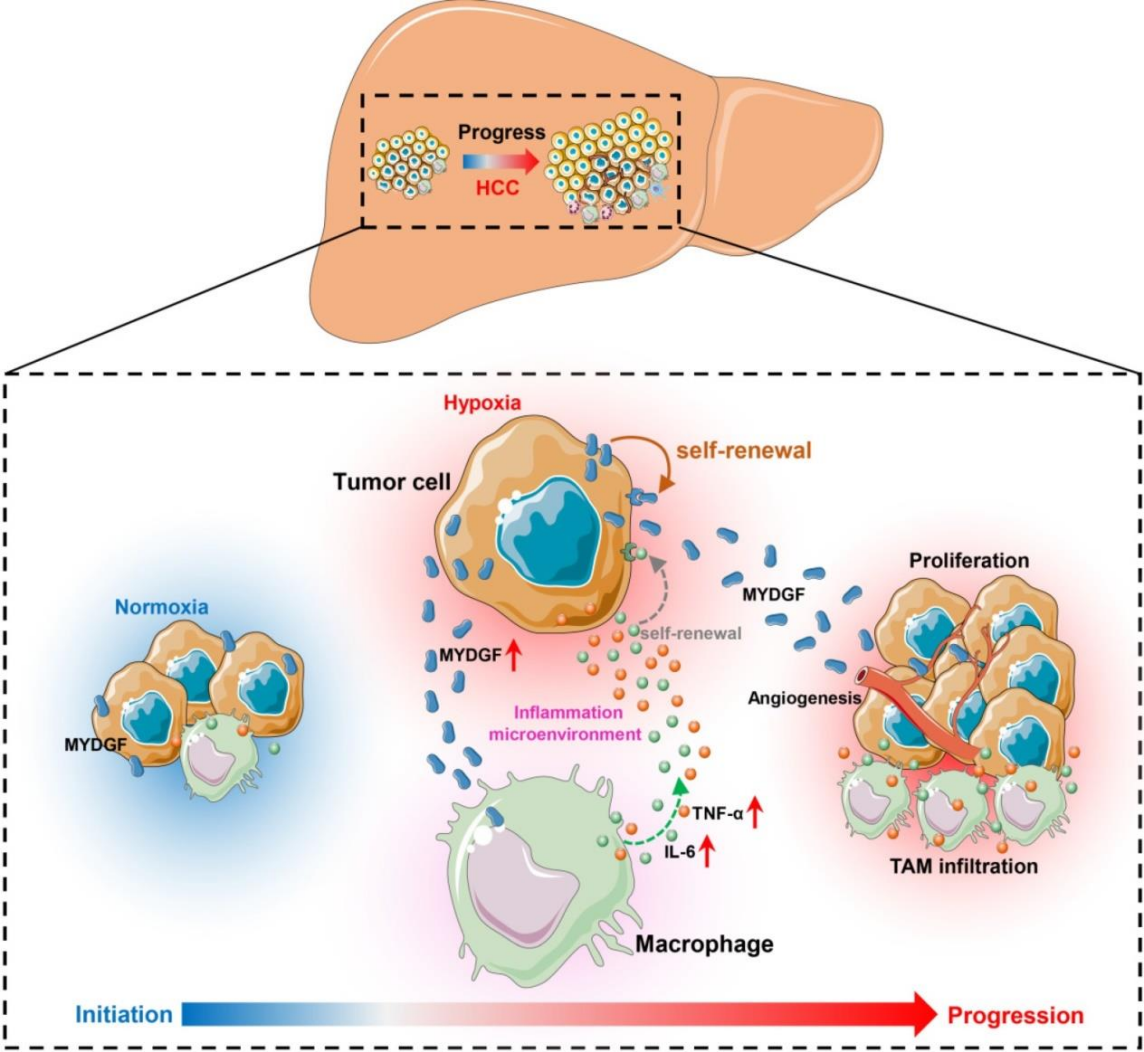
** Schematic illustration of the mechanisms by which MYDGF accelerates the progression of HCC.** At the initial state of tumor formation, tumor cells under a normal oxygen environment, the expression of MYDGF is relatively low. Under hypoxia condition, triggering the upregulation and release of MYDGF from the tumor cells, which could directly enhance self-renewal of hepatoma cells and promote proliferation of HCC through autocrine secretion. Meanwhile, MYDGF could also promote tumor angiogenesis. Through paracrine action, it could chemotactic macrophages and promote macrophages releasing various inflammatory cytokines, such as IL-6 and TNF-α. In this way, it may indirectly remodel the tumor microenvironment and accelerate the malignant progression of HCC.

Here, we combined bioinformatics and experimental methods to investigate the relationship between MYDGF and HCC progression. Taken together, we found MYDGF has multiple functions in HCC malignant progression. We first observed that MYDGF could be induced by hypoxia environment, which could be reversed by HIF inhibitor. MYDGF-mediated cell proliferation may be linked to its promotion of liver cancer stem cells (CSCs) self-renewal. Additionally, MYDGF could induce the chemotaxis of macrophages into tumor tissues and promote them releasing inflammatory cytokines, including IL-6 and TNF-α. Disordered inflammatory cytokines could remodel the tumor immune microenvironment, promote tumor angiogenesis, and accelerate the malignant progression of tumors (Figure 7).

HCC is highly malignant and metastasizes readily at the early stages. Its early diagnosis and prognostic evaluation are quite significant to patients. Imaging and the detection of serum markers are fundamental methods of identification in asymptomatic patients with HCC. However, at present no single diagnostic method is able to meet the sensitivity and specificity criteria required. In terms of serum markers, α-fetoprotein (AFP) is the preferred serum marker for the diagnosis and monitoring of HCC but it is negative in 40% patients with early stage HCC. Even in advanced HCC, the concentrations of AFP may be normal in 15-30% of patients 38. As a secreted protein, whether MYDFG could be used for early diagnosis of HCC is quite meaningful. Receiver operating characteristic (ROC) curve analysis revealed that MYDGF is a highly sensitive and specific biomarker for HCC samples to distinguish, and that it may even perform better than the clinically-used alpha-fetoprotein (AFP) from gene expression level (Figure S1F). Certainly, it is necessary to combine the expression of MYDGF in patient blood samples with clinical information for further analysis. Moreover, our work also revealed that MYDGF could be a prognostic marker for HCC.

There were still many shortcomings and deficiencies in this work, and further research is needed. The specific molecular mechanism of how MYDGF promotes CSCs self-renewal or TME disorder needs further exploration. Furthermore, its receptor is currently unknown. Identifying its receptor is critical for the development of targeted therapy. In conclusion, our study revealed that MYDGF plays an essential role in the progression of HCC. For HCC itself or the tumor microenvironment, MYDGF might be a potential therapeutic target.

## Materials and Methods

### Antibodies and Reagents

Recombinant human SF20 protein (Cat No. ab86915) and anti-SF20 antibody (Cat No. ab187919) were purchased from Abcam. Recombinant murine SF20 (Cat No. 210-25), human FGF (Cat No. 100-18B), human EGF (Cat No. GMP100-15), M-CSF (Cat No. 315-02) were acquired from PeproTech. Endothelial cell media (Cat No. 1001) was purchased from ScienCell. Mouse IL-6 (Cat No. 1210602), MCP-1 (Cat No. 1217392) and TNF-α (Cat No. 1217202) Elisa detection kits were purchased from DaKeWei. Bay 87-2243 (Cat No. S7309) was purchased from Selleck. Anti-NF-κB (Cat No. ab16502), anti-JNK (Cat No. ab179461), anti-Phospho-p44/42 MAPK (Erk1/2) (Thr202/Tyr204) (Cat No. ab17942), anti-CD31 (Cat No. ab134168), anti-Ki67 (Cat No. ab15580), anti-PCNA (Cat No. ab92552), and Anti-beta Actin (Cat No. ab8226) antibodies were purchased from Abcam. Anti-p-STAT3 (Tyr705) (Cat No. 9145), anti-STAT3 (Cat No. 9139) and anti-F4/80 (Cat No. 70076) were purchased from Cell Signaling Technology.

### Animals and tumor implantation

C57BL/6 mice were purchased from Beijing Vital River Laboratory Animal Technology Co., Ltd and housed in Specific Pathogen Free (SPF) environment with a 12/12 h day/night cycle. Xenografts were generated by subcutaneously injecting 2×10^6^ Hepa1-6-NC or Hepa1-6-shMYDGF cells suspended in 0.1 mL of Matrigel (Corning, Cat. No. 354234) into the flanks of the mice. Tumor volume was calculated using the formula: (length×width^2^)/2. All animal experiment protocols were reviewed and approved by the Institutional Animal Care and Use Committee of New Drug Evaluation and Research Center (China Pharmaceutical University, China).

### Clinical sample preparation

Fresh hepatocellular carcinoma tissues were obtained from Nanjing Drum Tower Hospital (Nanjing, China). The tissues were fixed in 10% formalin for 7 days at room temperature. Samples were then dehydrated, paraffin-embedded and sectioned at 4 μM for staining. The studies were approved by the ethical committee of Nanjing Drum Tower Hospital (Nanjing, China).

### Bioinformatics

First, the Oncomine^TM^ database (https://www.oncomine.org/) was utilized to analyze MYDGF expression in HCC and normal tissues. The threshold was set as *p* ≤ 0.01, 1.5-fold change and the top 10% of the genes. The parameters evaluated included p value and fold change. TCGA and GEO datasets were used to validate the relationship between MYDGF expression levels and HCC patient prognosis. IHC images of MYDGF protein expression level were obtained from the Human Protein Atlas database (https://www.proteinatlas.org/). GSEA analysis was performed as previous described 39. Liver cancer gene expression data (LIHC) in FPKM format was downloaded from TCGA database (https://portal.gdc.cancer.gov/). Association between gene expression and biological processes was analyzed using GSEA. The samples were ranked based on MYDGF expression level. The first 10% of the samples were classified as low MYDGF expression group, and the latter 10% were regarded as MYDGF high expression group. Software default settings were used and the significance threshold determined by permutation analysis (1000 permutations). A gene set with FDR < 0.25 was considered enriched between the 2 phenotypes under comparison. P-value and normalized enrichment score (NES) are used to rank the enriched pathways in each phenotype. The hallmark, KEGG, GO and oncogenic signatures from the Molecular Signatures Database (MsigDB, http://software.broadinstitute.org/gsea/index.jsp) were used for enrichment analysis.

### Cell culture

Hepa1-6, HepG2, HCCLM3, MHCC97H, BEL-7404 and SNU387 were purchased from Nanjing Kezhen Biological Co., Ltd (Nanjing, China). Human primary hepatic carcinoma cell line T1115 was established from a HCC patient and provided as gifts by Professor Cheng Qian from Third Military Medical University. Hepa1-6, HepG2, MHCC97H and BEL-7404 were cultured in DMEM (Biological Industries, Cat. No. 01-052-1A) supplemented with 10% FBS (Biological Industries, Cat. No. 04-001-1A) and 1% pen/strep (Hyclone, Cat. No. SV30010). SNU387 was cultured in RPMI 1640 (Biological Industries, Cat. No. 01-100-1A) with 10% FBS and 1% pen/strep. HUVEC cells (Cat. No. CRL-1730) were purchased from ATCC and cultured in endothelial cell medium supplemented with ECGS (ScienCell, Cat. No. 1001) and 5% FBS. All cells were grown in a humified incubator at 37 °C, 5% CO_2_.

### siRNA transfection

siRNAs targeting mouse MYDGF were purchased from GenePharma (Shanghai, China). The sequences are displayed in Supplementary Table 1. siRNA transfection was done using jetPRIME transfection reagent (Cat. No. 114-15) following manufacturer's instructions.

### Quantitative PCR

Reverse transcription and qPCR were done using HiScript II Q RT SuperMix for qPCR (+gDNA wiper) (Takara, Cat. No. R223-01) and Cham Q^TM^ Universal SYBR qPCR Master Mix (Takara, Cat. No. Q711-02) respectively. Primer sequences were listed in Supplementary Table 2. Data were normalized to GAPDH mRNA expression using the ^∆∆^Ct method.

### Cell viability assay

For cell viability assay, cells were seeded into 96-well plates at a density of 0.7 × 10^4^ cells per well. Cell viability was measured using the Sulforhodamine B (SRB) assay, as previously described 40, 41.

### Sphere formation assay

Sphere formation assays were performed as described previously 42, 43. Briefly, 3000 cells were seeded onto Costar ultra-low adhesion 6-well plates (Corning, Cat. No. 3471) and maintained in DMEM/F12 (Biological Industries, Cat. No. 01-170-1A) with 2% B27 (Gibco, Cat. No. 17504044), 100 U/mL penicillin, 100 μg/mL streptomycin, 20 ng/mL EGF and 20 ng/mL basic FGF. Individual spheres ≥ 100 μm from each replicate well (n ≥ 3) were counted under an inverted microscope at 40X magnification using Image-Pro Plus program (Media Cybernetics).

### Tube formation assays

A 24-well plate was coated with 250 μL matrigel™ (Corning, Cat. No. 354234) and incubated at 37 °C for 30 min. 6 × 10^4^ HUVECs cells, suspended in 250 μL of endothelial cell medium (ScienCell, Cat. No. 1001) with different concentrations of recombinant human MYDGF protein were then seeded. After 8-hour incubation at 37 °C, tube formation was examined under a microscope and complete tubular structures counted. 'Total Branching length' and 'Total meshes area' were analyzed using Image J's *'Angiogenesis'* plugin.

### Flow cytometry

The tissues were minced with gentle MACSTM Octo Dissociator (Miltenyi Biotec, Germany) and digested in RPMI-1640 media (Biological Industries, Israel) containing 0.25 mg/mL Collagenase D (Roche, Mannheim, Germany), 0.1 mg/mL DNase I (Sigma, USA). The digested cell suspension was then washed with PBS and filtered through a 70 μm cell strainer. Cells were stained with fluorescently conjugated antibodies: LIVE/DEAD™ Fixable far-red dead cell stain kit (Invitrogen, Cat. No. L10119), LIVE/DEAD™ Fixable Violet dead cell stain kit (Invitrogen, Cat. No. L34964), CD45 (APC-Cy7, Clone: 30-F11, Biolegend, Cat. No. 103116), CD11b (BV510, Clone: M1/70, Biolegend, Cat. No. 101263), F4/80 (APC, Clone: BM8, Biolegend, Cat. No. 123116), Ly6C (PE, Clone: HK1.4, Biolegend, Cat. No. 128008, Ly6G (PE-CY7, Clone: 1A8, Biolegend, Cat. No. 127618). CD45 (BV510, Clone: 30-F11, Biolegend, Cat. No. 103138), CD3e (PE-CY7, Clone: 145-2C11, Biosciences, Cat. No. 552774), CD8 (PE, Clone: 53-6.7, Biolegend, Cat. No. 100708), PD1 (APC, Clone: RMP1-30, Biolegend, Cat. No. 109112), TIM3 (BV421, Clone: RMT3-23, Biolegend, Cat. No. 119723). Cells were detected by the BD FACSVerse^TM^ Flow Cytometer (BD, USA) and were analyzed by FlowJo Software.

### BMDM and M2 type macrophages culture

Femur and tibia freshly isolated from C57BL/6 mice were flushed with PBS. Cells were cultured in DMEM supplemented with 10% heat inactivated FBS and 20 ng/mL M-CSF for 6 days for differentiation into Bone marrow-derived macrophages (BMDM). For M2 type macrophages differentiation, 10 ng/mL IL-4 was added into mature BMDM 44. On day 7, BMDMs and M2 type macrophages were seeded onto plates and treated with 100 ng/mL murine recombinant MYDGF as indicated in the legend. After 48 h, the cell supernatant was collected for Elisa detection.

### Macrophage chemotaxis assay

Transwells with 8 μm were used as the upper chamber, 7 × 10^5^ macrophages suspended in serum-free medium were added into the upper chamber. BMDM supernatant treated with 100 ng/mL MYDGF were added in the lower chamber for 24 h. The upper chamber was then fixed with 4% paraformaldehyde and stained before examination and imaging of migrated macrophages.

### Statistical analysis

All data analyses were done using GraphPad Prism 8 or SPSS version 19. Data are presented as mean ± SEM. Two-tailed student's *t* test was performed when only 2 groups were compared. One-way ANOVA followed by Dunnett's posttests were used to compare differences between multiple groups unless otherwise stated. Kaplan-Meier curve was used to compare HCC patient survival.^ *^P < 0.05, ^**^P < 0.01, ^***^P < 0.001.

## Supplementary Material

Supplementary figures and tables.

## Abbreviations

HCC: Hepatocellular carcinoma
MYDGF: Myeloid-Derived Growth Factor
TCGA: Cancer Genome Atlas
GSEA: Gene Set Enrichment Analysis
OS: Overall Survival
ROC: Receiver Operating Characteristic
CSCs: Cancer Stem Cells
TAM: tumor-associated macrophage
BMDM: Bone Marrow Derived Macrophage
M-CSF: Macrophage colony stimulating factor
TAM: Tumor-Associated Macrophage
TME: Tumor microenvironment
AFP: α-fetoprotein
