# Supplementary material for: Hypoxia-induced myeloid derived growth factor promotes hepatocellular carcinoma progression through remodeling tumor microenvironment
Source: Theranostics. 2021 Jan 1;11(1):209–21. doi: 10.7150/thno.49327 (PMC7681097; doi:10.7150/thno.49327)
Supplement: Supplementary file 1 — Supplementary figures and tables. [file thnov11p0209s1.pdf]

Supplementary Figures

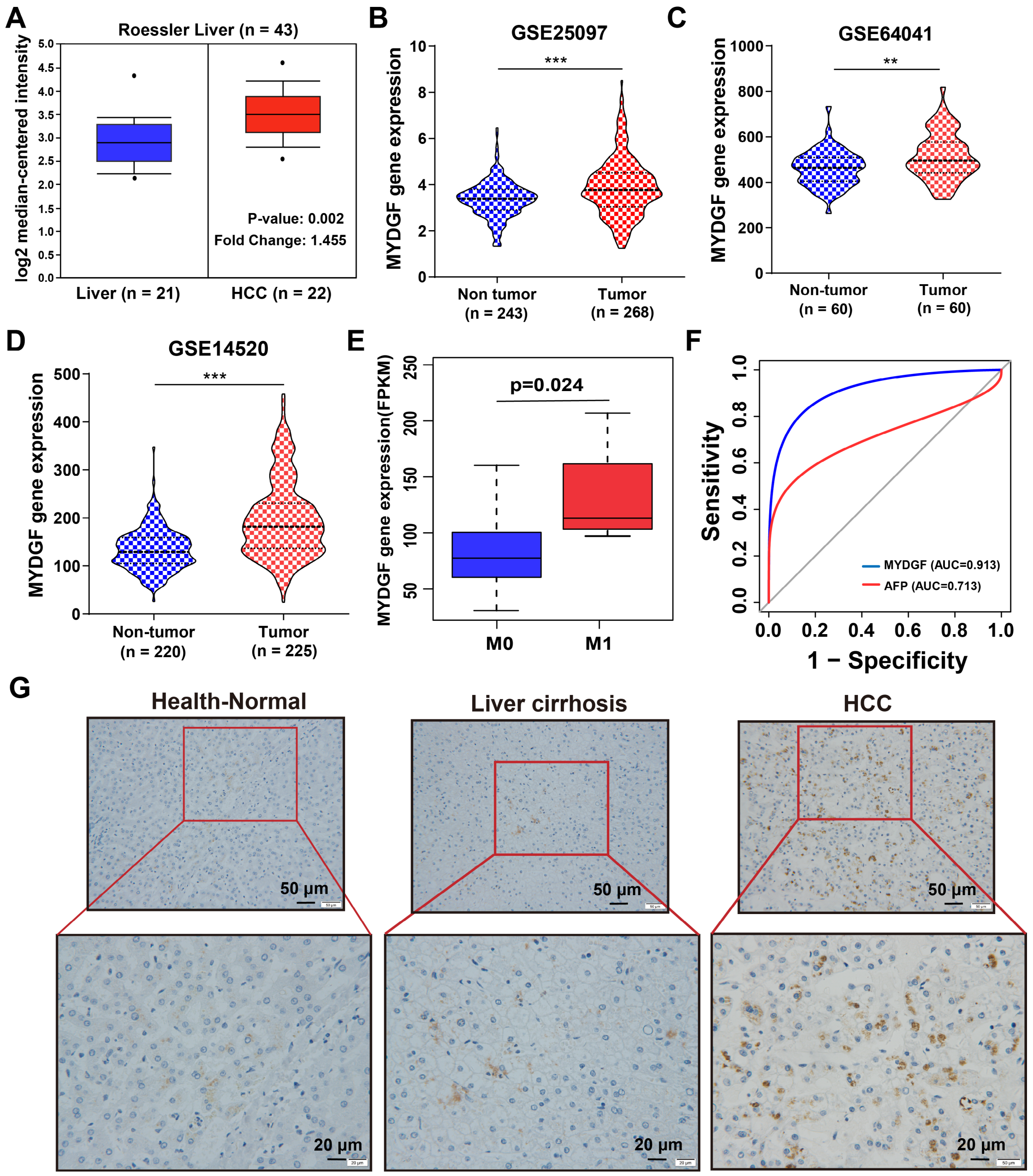

Figure S1. MYDGF is upregulated in HCC. (A) MYDGF gene expression in Roessler liver dataset from Oncomine database, containing 43 samples. (B) GSE25097 dataset from GEO database, which contains 243 non-tumor samples and 268 HCC tumor samples. (C) GSE64041 dataset from GEO database which includes 60 paired non-tumor and HCC tumor samples. (D) GSE14520 dataset from GEO database, it includes 220 non-tumor samples and 225 HCC tumor samples. (E) Gene

expression of MYDGF in no-distant metastasis (M0) and distant metastasis (M1) samples. (F) The ROC curve compared the ability of MYDGF to distinguish normal tissues from HCC samples at the genetic level. (G) Immunohistochemistry results showed that MYDGF expression is negative in normal liver tissue and cirrhosis samples, but specifically positive stain can be found in Stage I hepatocellular carcinoma samples.

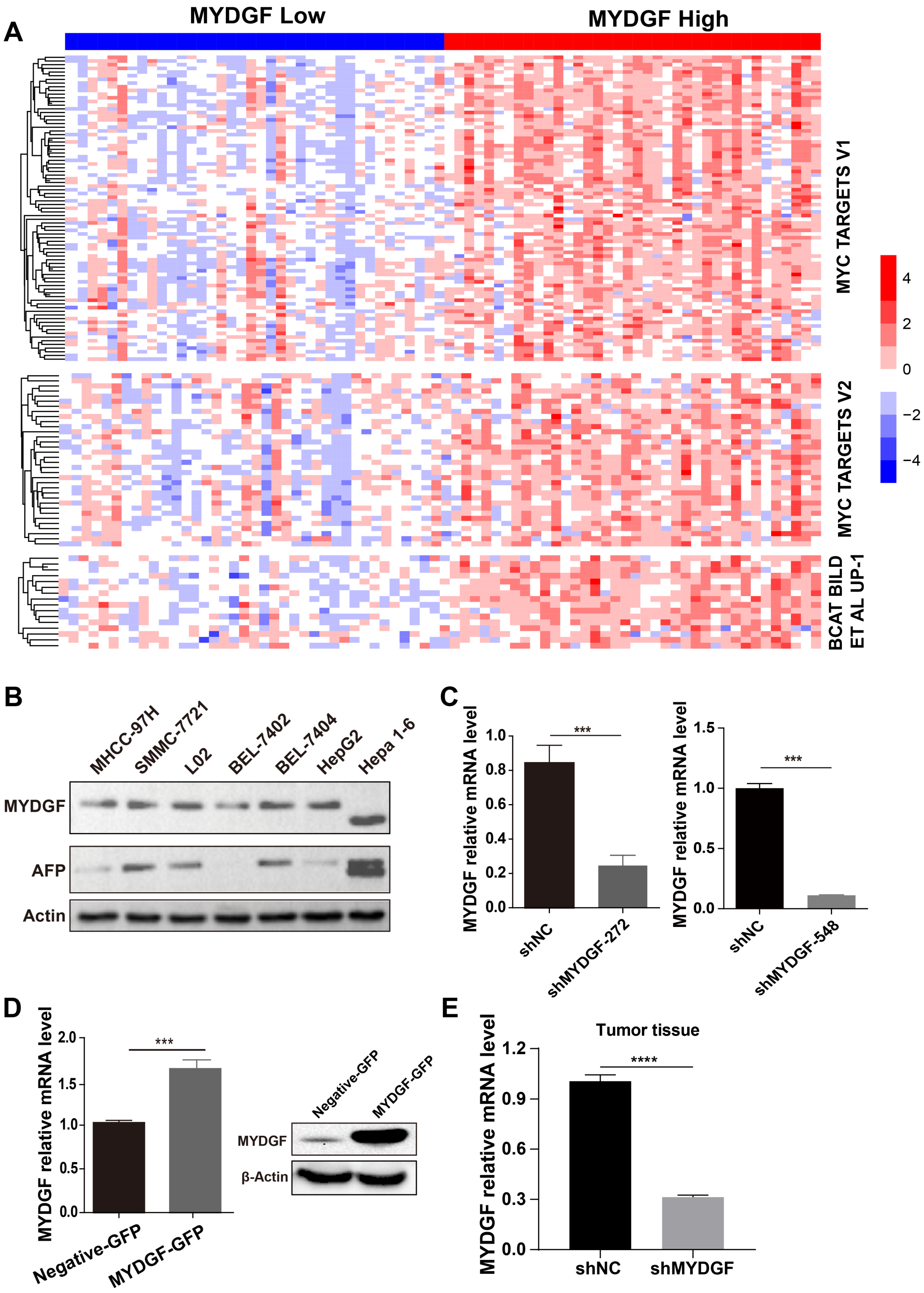

Figure S2. MYDGF promotes cell proliferation *in vitro*. (A) MYDGF expression in high and low groups of enriched signatures, include "HALLMARK_MYC_TARGET_V1", "HALLMARK_MYC_TARGET_V2" and "BCAT_BILD_ET_AL_UP-1". (B) MYDGF and AFP protein expression in 6 HCC

cancer cell lines and a normal liver cell line LO2. (C) Two sequences with the highest knockdown efficiency were selected for packaging lentiviruses for the construction of stable cell lines. The knockdown efficiency of MYDGF in Hepa1-6 cells is shown. (D) BEL-7404 was used to construct MYDGF overexpressing cell lines, and the efficiency of MYDGF overexpression was tested. (E) The expression level of MYDGF in the xenograft tumor tissue at the end of the experiment.

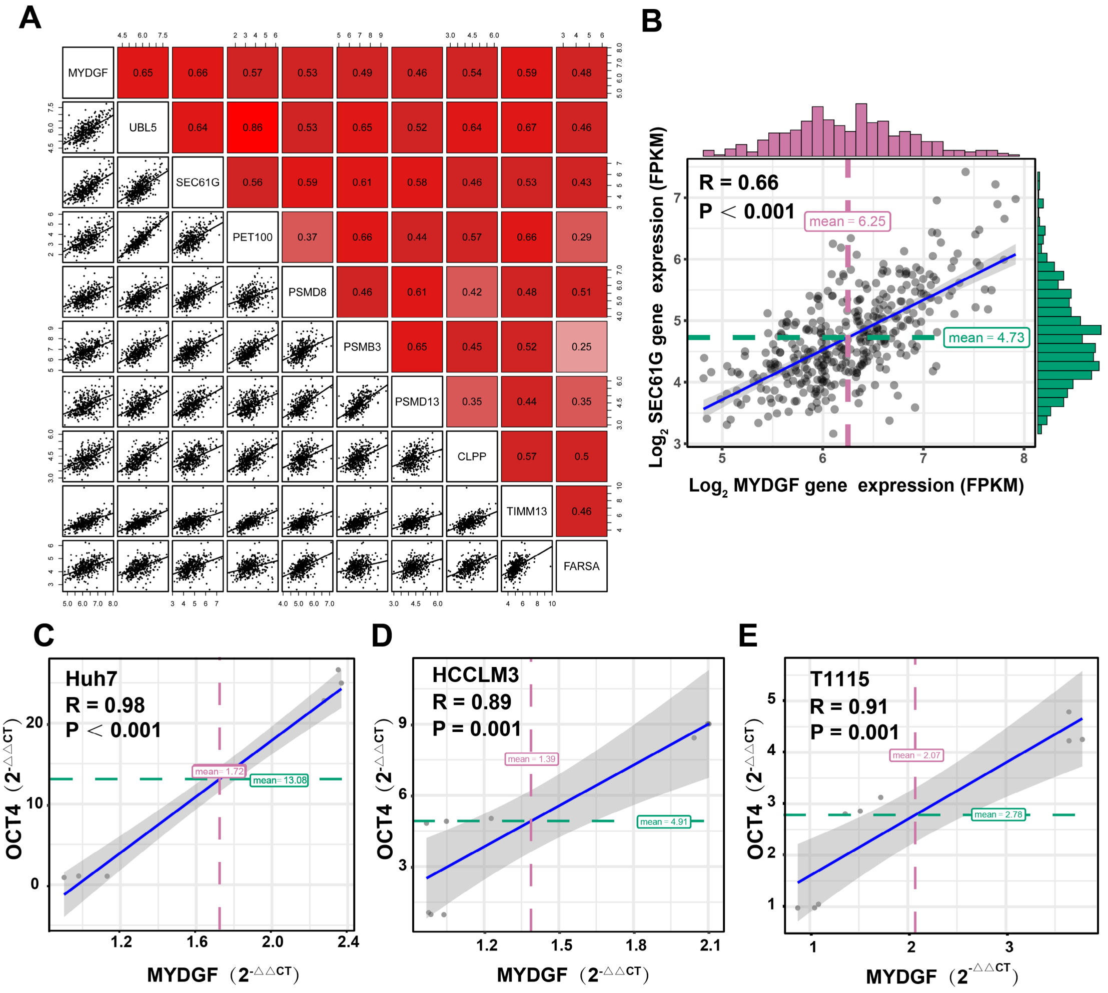

Figure S3. MYDGF expression is associated with stem cell related signatures and genes. (A) Correlation between MYDGF and top three leading-edge genes from each stem cell related signatures in TCGA database. (B) The Most relevant gene was shown in the scatter plot. Correlation value was calculated using person method. (C-E) Correlation between MYDGF and stem cell marker OCT4 in three human HCC cell lines (Huh7, HCCLM3, T1115).

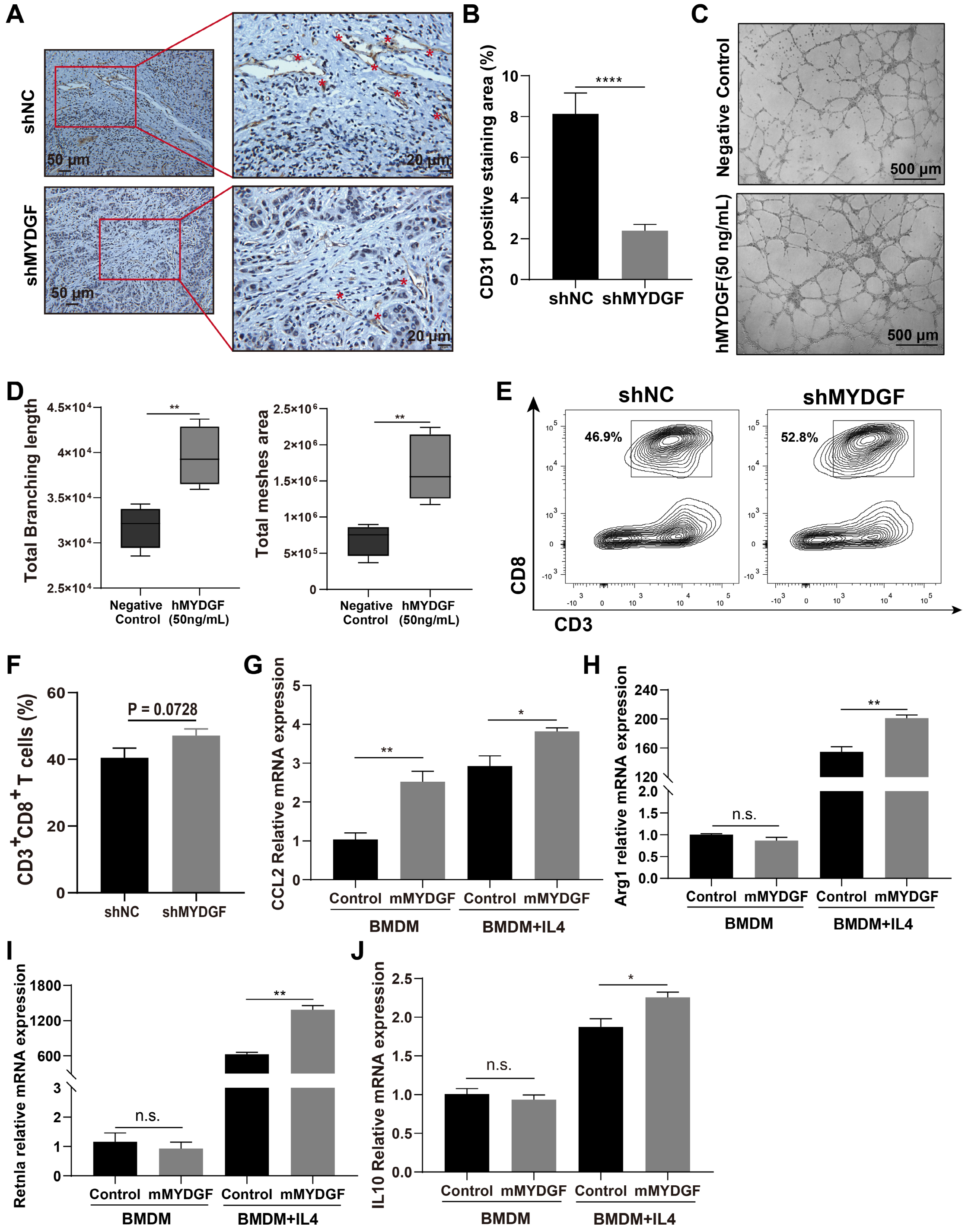

Figure S4. MYDGF indirectly promoted HCC development by remodeling TME. (A) Expression of CD31 in subcutaneous tumors of C57BL/6 mice was determined using immunohistochemical staining, the scale bars are shown in the figures. (B) CD31 positive staining area fraction was quantified by Image J software. (C) Tube formation assay was performed using HUVEC cells cultured with 50 ng/mL human

MYDGF recombinant protein for 8 hours. Cells were examined using a Leica microscope. (D) Total tube length and total meshes area was calculated by Image J software. (E) The proportion of $CD3^{+}CD8^{+}$ T cells infiltration in tumor tissue was detected by flow cytometry. (F) Quantitative statistics of $CD3^{+}CD8^{+}$ T cells infiltration fraction. (G) CCL2 mRNA expression in BMDM and M2 type macrophages cultured with 100 ng/mL MYDGF recombinant protein. (H-J) Arg1, Retnla and IL-10 mRNA level in BMDM and M2 type macrophages cultured with 100 ng/mL MYDGF recombinant protein.

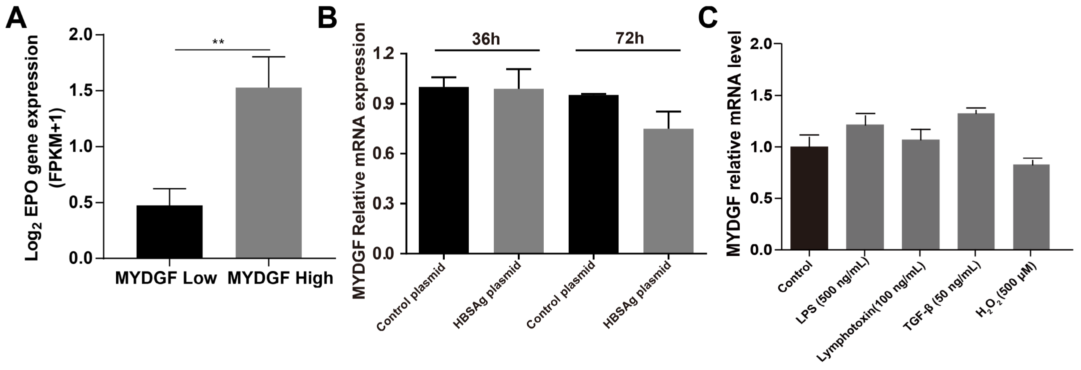

Figure S5. Hypoxia induces MYDGF expression in HCC. (A) EPO gene expression in $MYDGF^{high}$ and $MYDGF^{Low}$ group from the "CELLULAR RESPONSE TO HYPOXIA" signature. (B) HepG2 cells were transfected with HBS-Ag plasmid and the mRNA expression of MYDGF at 36 hours and 72 hours was detected. (C) HepG2 cells were cultured with LPS, lymphotoxin, TGF-β and H_2O_2 to simulate the factors that accelerate the progression of HCC in the microenvironment.

Table 1. MYDGF siRNA sequences

MYDGF(Mus)	Sequences	
	Sense (5'-3')	Antisense (5'-3')
siMYDGF-177	CCCGGGAACAAGUUUACAUTT	AUGUAAACUUGUUCCCGGGTT
siMYDGF-272	GCACUUUACCUGUACCAUCTT	GAUGGUACAGGUAAAGUGCTT
siMYDGF-351	GCUGAGAUCGAGUAUGCCATT	UGGCAUACUCGAUCUCAGCTT
siMYDGF-226	CCAACGAGCAAUGGCAGAUTT	AUCUGCCAUUGCUCGUUGGTT
siMYDGF-468	GCCUUCAAAGCUGAGCUCUTT	AGAGCUCAGCUUUGAAGGCTT
siMYDGF-548	CCUUCAUGUCCACGUUCCUTT	AGGAACGUGGACAUGAAGGTT

Table 2. The primer sequences used for qPCR

Genes	Sequence (5' to 3')
hMYDGF-F	CTGAAGTGAGTCCGGGAGC
hMYDGF-R	TGTTTGGGTTGGAGTTTGC
mMYDGF-F	ACATGTACATTCACCTACGCTT
mMYDGF-R	TGGTACAGGTAAAGTGCTGGC
mTNF-F	CCCTCACACTCAGATCATCTTCT
mTNF-R	GCTACGACGTGGGCTACAG
mIL-6-F	TAGTCCTTCCTACCCCAATTTCC
mIL-6-R	TTGGTCCTTAGCCACTCCTTC
mCCL2-F	TTAAAAACCTGGATCGGAACCAA
mCCL2-R	GCATTAGCTTCAGATTTACGGGT
mIL-10-F	GCTCTTACTGACTGGCATGAG
mIL-10-R	CGCAGCTCTAGGAGCATGTG
mArg1-F	CTCCAAGCCAAAGTCCTTAGAG
mArg1-R	AGGAGCTGTCATTAGGGACATC
mRetnla-F	CCAATCCAGCTAACTATCCCTCC
mRetnla-R	ACCCAGTAGCAGTCATCCCA
hGAPDH-F	CCTTCATTGACCTCAACTAC
hGAPDH-R	GAAGATGGTGATGGGATTTC
mGAPDH-F	AGTGGCAAAGTGGAGATT
mGAPDH-R	GTGGAGTCATACTGGAACA
